# *ITGA3* and *ITGB4* expression biomarkers estimate the risks of locoregional and hematogenous dissemination of oral squamous cell carcinoma

**DOI:** 10.1186/1471-2407-13-410

**Published:** 2013-09-05

**Authors:** Masaki Nagata, Arhab A Noman, Kenji Suzuki, Hiroshi Kurita, Makoto Ohnishi, Tokio Ohyama, Nobutaka Kitamura, Takanori Kobayashi, Kohya Uematsu, Katsu Takahashi, Naoki Kodama, Tomoyuki Kawase, Hideyuki Hoshina, Nobuyuki Ikeda, Susumu Shingaki, Ritsuo Takagi

**Affiliations:** 1 Division of Oral and Maxillofacial Surgery, Niigata University Graduate School of Medical and Dental Sciences, Gakkocho-dori 2-5274, Chuo-ku, Niigata 951-8514, Japan; 2 Department of Gastroenterology, Niigata University Graduate School of Medical and Dental Sciences, Asahimachi-dori 1-757, Chuo-ku, Niigata 951-8510, Japan; 3 Department of Dentistry and Oral Surgery, Shinshu University School of Medicine, Asahi 3-1-1, Matsumoto 390-8621, Japan; 4 Division of Dental Clinic and Oral Surgery, Nagaoka Red Cross Hospital, Terashimamachi 297-1, Nagaoka 940-2085, Japan; 5 Department of Medical Informatics, Niigata University Medical & Dental Hospital, Asahimachi-dori 1-754, Chuo-ku, Niigata 951-8520, Japan; 6 Department of Oral and Maxillofacial Surgery, Kyoto University Graduate School of Medicine, Shogoin-Kawahara-cho, Sakyo-ku, Kyoto 606-8507, Japan; 7 Division of Oral Bioengineering, Department of Tissue Regeneration and Reconstitution, Niigata University Graduate School of Medical and Dental Sciences, Gakkocho-dori 2-5274, Chuo-kuNiigata 951-8514, Japan; 8 Division of Reconstructive Surgery for Oral and Maxillofacial Region, Niigata University Graduate School of Medical and Dental Sciences, Gakkocho-dori 2-5274, Chuo-ku, Niigata 951-8514, Japan

**Keywords:** Squamous cell carcinoma, Biomarker, Metastasis, Integrin alpha3, Integrin beta4

## Abstract

**Background:**

Molecular biomarkers are essential for monitoring treatment effects, predicting prognosis, and improving survival rate in oral squamous cell carcinoma. This study sought to verify the effectiveness of two integrin gene expression ratios as biomarkers.

**Methods:**

Gene expression analyses of integrin α3 *(ITGA3),* integrin β4 (*ITGB4*), CD9 antigen (*CD9*), and plakoglobin (*JUP*) by quantitative real-time PCR were conducted on total RNA from 270 OSCC cases. The logrank test, Cox proportional hazards model, and Kaplan-Meier estimates were performed on the gene expression ratios of ITGA3/CD9 and ITGB4/JUP and on the clinicopathological parameters for major clinical events.

**Results:**

A high rate (around 80%) of lymph node metastasis was found in cases with a high ITGA3/CD9 ratio (high-ITGA3/CD9) and invasive histopathology (YK4). Primary site recurrence (PSR) was associated with high-ITGA3/CD9, T3-4 (TNM class), and positive margin, indicating that PSR is synergistically influenced by treatment failure and biological malignancy. A high ITGB4/JUP ratio (high-ITGB4/JUP) was revealed to be a primary contributor to distant metastasis without the involvement of clinicopathological factors, suggesting intervention of a critical step dependent on the function of the integrin β4 subunit. Kaplan-Meier curves revealed positive margin as a lethal treatment consequence in high-ITGA3/CD9 and YK4 double-positive cases.

**Conclusion:**

Two types of metastatic trait were found in OSCC: locoregional dissemination, which was reflected by high-ITGA3/CD9, and distant metastasis through hematogenous dissemination, uniquely distinguished by high-ITGB4/JUP. The clinical significance of the integrin biomarkers implies that biological mechanisms such as cancer cell motility and anchorage-independent survival are vital for OSCC recurrence and metastasis.

## Background

Around 260 000 new cases of oral cancer in the tongue, gingiva, oral floor, lip, and buccal mucosa are reported annually worldwide, and deaths from the disease reach approximately 127 000 [1]. Squamous cell carcinoma of the oral cavity (OSCC) is the most prevalent malignancy of the head and neck region. Despite recent improvements in treatment, the survival of OSCC patients has not improved greatly over the past few decades [2]. Treatment failures of OSCC are primarily due to local and regional recurrence, and uncontrollable deaths can occur from distant metastasis [3,4], It is particularly important, therefore, to ensure a sufficient resection margin that takes the degree of infiltration into consideration [4-7]. While the rate of distant metastasis is less than 5% [7,8], there is no curable treatment once metastatic foci become visible. Another issue associated with OSCC treatment is a decline in the quality of life (QOL) of the patients because of unavoidable stomatognathic dysfunction [9]. To improve the survival rate and patient QOL, it is essential to fully understand the risks of locoregional recurrence and distant metastasis. Histopathological features, immunohistological markers, blood biomarkers, and clinical features have been used as prognostic factors [10-12]; however, these parameters cannot provide relevant information during the early phases of treatment. Therefore, recent attempts to improve the diagnostic system have focused on gene mutations or polymorphisms and altered expression levels of biomarkers [13].

In our previous studies, we used microarray analysis and reverse transcription quantitative real time polymerase chain reaction (RT-QPCR) to report the potential use of integrin and tetraspanin family molecules as biomarkers for OSCC malignancy [14-16]. The integrin (ITG) molecule functions as a cell surface receptor that mediates extracellular mechanical and chemical signals into the cell interior, which modulates different signal transduction cascades. ITG also coordinates cell survival, apoptosis, proliferation, and motility and influences cell differentiation [17-20]. In the present study, we used RT-QPCR to determine the gene expression of integrin α3 (*ITGA3*) and integrin β4 (*ITGB4*), as well as *CD9* and plakoglobin (*JUP*), a desmosomal anchor protein gene [15]. We report here that certain *ITGA3/CD9* and *ITGB4/JUP* gene expression ratios are specifically related to individual clinical events such as lymph node metastasis, primary site recurrence, distant metastasis, and uncontrollable death from OSCC.

## Methods

### Patients and specimens

Tumor samples for gene expression analyses were collected at the time of biopsy from 270 patients with OSCC who were treated at the Dental Department of Niigata University Medical and Dental Hospital, Niigata, Japan, the Special Dental Care and Oral Surgery of Shinshu University Hospital, Nagano, Japan, and the Division of Oral Surgery of Nagaoka Red Cross Hospital, Nagaoka, Japan from 1999 to 2008 (Table 1). The treatment modalities included local resection, composite resection (resection of a primary oral cancer, a portion of the oral floor and mandible, and reconstruction with tissue transplantation and neck dissection), and composite resection with radiation therapy with or without intravenous adjuvant chemotherapy.

**Table 1:** Clinicopathological data of 270 patients with oral squamous cell carcinoma

**Clinicopathological factor**		**No. of patients (%)**
	Total	270
Observation period (days)	61-2182 (average, 1253.79)	
Age (years)	21 - 92 (average, 66.70)	
Sex	Male	166 (61.48)
	Female	104 (38.52)
Tumor size (mm)^1^	5-60 (average, 26.63)	
	≤20	86 (31.85)
	21-30	97 (35.93)
	31-40	62 (22.96)
	>40	25 (9.26)
Tumor status^2^	T1	83 (30.74)
	T2	123 (45.56)
	T3	5 (1.85)
	T4	59 (21.85)
Lymph node metastasis^3^	pN0	149 (55.19)
	pN1	41 (15.18)
	pN2	80 (29.63)
	pN3	0 (0)
Histologic grade (YK4)^4^	1-3	115 (42.59)
	4c-d	155 (57.41)
Surgical margin^5^	Negative	244 (90.37)
	Positive	26 (9.63)
Primary site recurrence	Negative	240 (88.89)
	Positive	30 (11.11)
Distant metastasis	Negative	256 (94.81)
	Positive	14 (5.19)
Death outcome^6^	Alive	233 (86.30)
	Dead	37 (13.70)

^1^Major width of the tumor. ^2^Tumor (T) category according to the International Union Against Cancer (UICC) TNM classification of malignant tumors of the lip and oral cavity. ^3^Lymph node (pN) category determined by pathologic examination of a surgical specimen, according to the UICC TNM classification of malignant tumors of the lip and oral cavity. ^4^Histopathologic classification of oral squamous cell carcinoma (YK grade) according to Yamamoto et al., 1983 (10). ^5^Histological tumor status of the surgical margin. ^6^Death outcome from uncontrollable oral squamous cell carcinoma.

This study was performed in accordance with the guidelines of the Declaration of Helsinki and the study protocol for this project was approved by the Research Ethics Committee of Niigata University Medical and Dental Hospital, the Ethics Committee of Shinshu University School of Medicine, and the Ethics Committee of Nagaoka Red Cross Hospital. A written letter of consent was processed after obtaining the patient informed consent to participate in this study.

### Total RNA extraction from carcinoma tissue

Cancer tissue specimens were preserved by immersion in RNAlater solution (Ambion Inc., Austin, TX, USA) immediately after sampling. The extraction of total RNA was performed using the RNeasy Lipid Tissue Mini Kit (QIAGEN, Tokyo, Japan) after homogenization by TissueLyser LT (QIAGEN) in QIAzol Lysis Reagent according to the manufacturer’s standard protocol. Synthesis of first-strand cDNA was performed by reverse transcription using total RNA (0.2–1 μg) as a template (Super Script III, Life Technologies, Carlsbad, CA, USA).

### Gene expression analysis by quantitative real-time polymerase chain reaction

RT-QPCR was performed on a Smart Cycler (Cepheid, Sunnyvale, CA, USA) using cDNA synthesized from the cancer specimens and TaqMan probes (TaqMan Gene expression Assays, Life Technologies) according to the following protocol: 600 s at 95°C, followed by thermal cycles of 15 s at 95°C and 60 s at 60°C for the extension. Relative standard curves representing several 10-fold cDNA dilutions (1:10:100:1 000:10 000:100 000) from an OSCC tissue sample were used for the linear regression analysis of other samples. The manufacturer’s TaqMan probe assay IDs are as follows: *ITGA3*: Hs00233722_m1; *ITGB4*: Hs01103172_g1; *CD9* [NM_001769]: Hs01124027_m1; and *JUP*: Hs00158408_m1.

### Histopathological classification of OSCC

**Figure 1:**
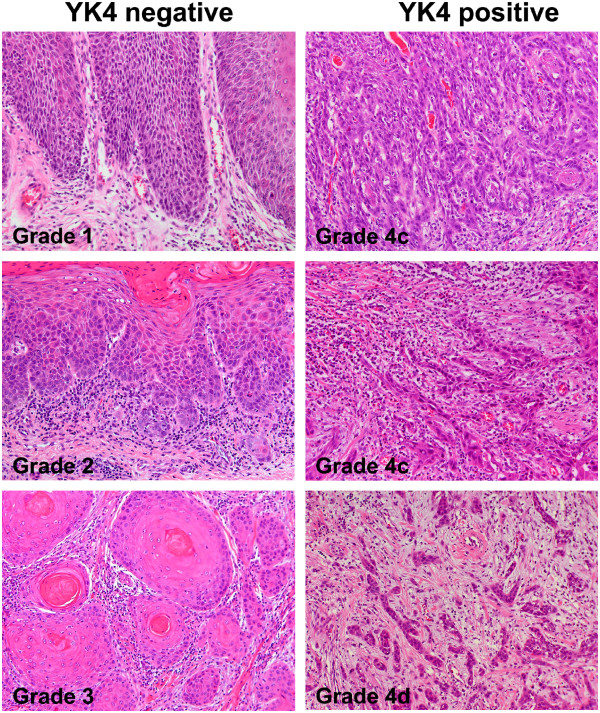
**Histopathological categorization by mode of invasion**[10]**.** Grade 1: well-defined borderline along the basal layer of squamous cell carcinoma (SCC). Grade 2: less-marked borderline with occasional growth of SCC cell groups. Grade 3: invasive growth of SCC cell groups with no distinct borderline. Grade 4c: diffuse invasion of cord-like SCC cells. Grade 4d: diffuse invasion of a single SCC cell or a few SCC cells in the deeper portion. Grade 1, 2, and 3 were categorized to YK4 negative (YK4-), and Grade 4c and 4d to YK4 positive (YK4+).

Hematoxylin and eosin staining was conducted using 10% formalin-fixed, paraffin-embedded sections of OSCC. Histopathological malignancy was examined based on the mode of invasion, as defined by a previous study [10] in which histopathological invasiveness was classified as Grade 1–4. We categorized YK4- as Grade 1–3 and YK4+ as Grade 4 (Figure 1).

### Statistical analysis

Two integrin gene expression ratios, ITGA3/CD9 and ITGB4/JUP, were calculated for each of the 270 patients with OSCC (Table 1). Clinicopathological parameters were age, sex, tumor size, T category (UICC TNM Classification of Malignant Tumors), histopathological mode of invasion (YK4) [10], and positive margin (histological tumor positive at the surgical margin). Clinical events were lymph node metastasis (LNM) determined by histopathological examination of the surgical specimen, primary site recurrence after surgery (PSR), distant metastasis after surgical excision of the primary cancer (DM), and death from uncontrollable OSCC (DO). DM following locoregional failure was included as locoregional recurrence.

The influence of the two integrin gene expression ratios and clinicopathological parameters on LNM, PSR, DM, and DO were reviewed by univariate analysis (logrank test) to optimize the combination of variables for the following multivariate analysis (Table 2). Analyses by Cox proportional hazards model and Kaplan-Meier curve were performed for LNM, PSR, and DO (or disease-specific survival) as endpoint events (SPSS 18.0, IBM Japan, Tokyo, Japan). Durations to the events were calculated from the date of first visit to the date of neck dissection, diagnosis of recurrence, and date of OSCC death or final observation. *P-*values ≤0.05 were assigned as the level of significance.

**Table 2:** Logrank test (Mantel-Cox)

	**Lymph node metastasis**	**Primary site recurrence**	**Distant metastasis**	**OSCC death **^**7**^
**Gene expression ratio**				
High-ITGA3/CD9	0.000	0.005	0.001	0.000
High-ITGB4/JUP	0.022	0.237	0.000	0.001
**Clinicopathological parameter**				
Age^1^	0.449	0.321	0.207	0.365
Sex	0.417	0.858	0.149	0.584
Size^2^	0.000	0.007	0.703	0.084
T3-4^3^	0.000	0.000	0.390	0.092
YK4^4^	0.000	0.018	0.001	0.000
Positive margin^5^	------	0.000	0.427	0.000
**Clinical event**				
Lymph node metastasis^6^	------	0.001	0.000	0.000
Primary site recurrence	------	------	0.840	0.000
Distant metastasis	------	------	------	0.000

Numbers in the table show P-values for the parameters. ^1^Age was categorized into two groups ≥69 and <69 based on the median age. ^2^Major width of the tumor was categorized into >30 mm and ≤30 mm. ^3^ T3 to T4 of tumor (T) category according to the International Union Against Cancer (UICC) TNM classification of malignant tumors of the lip and oral cavity. ^4^4c or 4d of the histopathologic classification of oral squamous cell carcinoma according to Yamamoto et al., 1983 (10). ^5^Histological tumor positive of the surgical margin. ^6^Determined by pathologic examination of a surgical specimen. ^7^Death outcome from oral squamous cell carcinoma. Continuous variables of gene expression ratios are categorized according to the cut off points introduced by receiver operating characteristic curves. Median of ITGA3/CD9 and higher 30% group of ITGB4/JUP are used for categorization.

## Results

### Univariate analysis

The results of univariate analysis by the logrank test revealed the parameters that influenced the three clinical events and death outcome (DO) in each column of Table 2. Lymph node metastasis (LNM) was significantly associated with a high ITGA3/CD9 ratio (high-ITGA3/CD9), tumor size and T3-4, which relate to the extent of tumor invasion, and histopathological mode of invasion (YK4). Primary site recurrence (PSR) was significantly associated with high-ITGA3/CD9, tumor size, T3-4 and positive margin, while distant metastasis was associated with high-ITGA3/CD9, a high ITGB4/JUP ratio (high-ITGB4/JUP), and YK4. DO was also associated with high-ITGA3/CD9 and high-ITGB4/JUP, and clinicopathological parameters with YK4 and positive margin.

### Lymph node metastasis

**Figure 2:**
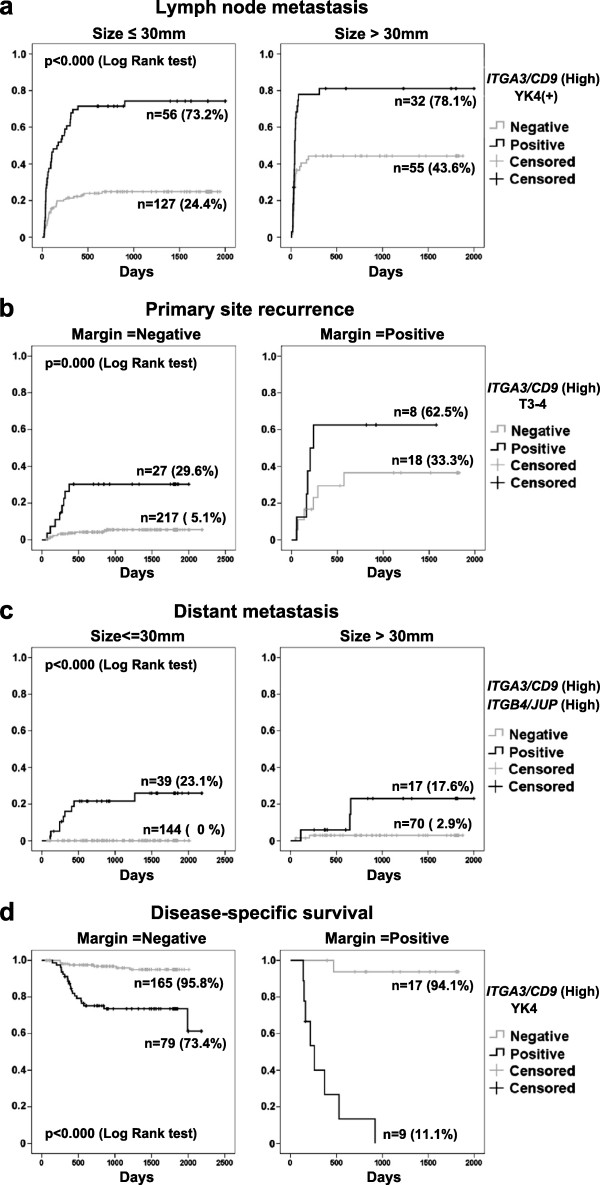
**Kaplan-Meier survival curves for 270 patients with oral squamous cell carcinoma.** Lymph node metastasis **(a)**, primary site recurrence **(b)**, distant metastasis **(c)**, and disease-specific survival **(d)**. Each consequence was stratified by a clinical event which effectively demonstrated influence of the factors. **(a)**-**(c)**: Curves show 1 minus cumulative survival. **(d)**: Curve shows cumulative survival.

In the Cox proportional hazards model of LNM, high-ITGA3/CD9, YK4, and the major width of the tumor (size) were reported as independently significant variables (Table 3A). The rate of LNM was represented by the *Kaplan-Meier (K-M) curve* (one minus cumulative survival) between groups of [high-ITGA3/CD9 and YK4]-positive cases and the remaining (negative) cases according to size category strata (Figure 2a). The [high-ITGA3/CD9 and YK4]-positive cases consistently exhibited a higher rate of LNM (around 80%) irrespective of the size strata, but the negative cases revealed an increasing rate of LNM with larger tumor size (>30 mm).

**Table 3:** Cox proportional hazards model

**A**	**Lymph node metastasis**						
Variable	B	SE	Wald	*P*	OR	95% CI for OR	
						Lower limit	Upper limit
High-ITGA3/CD9	1.063	0.203	27.545	0.000	2.896	1.947	4.307
YK4^1^	0.961	0.216	19.887	0.000	2.614	1.714	3.989
Size^2^	0.622	0.186	11.126	0.001	1.862	1.292	2.684
**B**	**Primary site recurrence**						
Variable	B	SE	Wald	*P*	OR	95% CI for OR	
						Lower limit	Upper limit
High-ITGA3/CD9	1.090	0.415	6.895	0.009	2.973	1.318	6.706
T3-4^3^	0.955	0.387	6.088	0.014	2.597	1.217	5.544
Positive margin^4^	1.715	0.402	18.183	0.000	5.556	2.526	12.221
**C**	**Distant metastasis**						
Variable	B	SE	Wald	*P*	OR	95% CI for OR	
						Lower limit	Upper limit
High-ITGB4/JUP	2.311	0.771	8.990	0.003	10.088	2.227	45.704
High-ITGA3/CD9	2.108	1.047	4.051	0.044	8.233	1.057	64.135
**D**	**Death outcome**^**5**^						
Variable	B	SE	Wald	*P*	OR	95% CI for OR	
						Lower limit	Upper limit
High-ITGA3/CD9	1.618	0.450	12.947	0.000	5.041	2.089	12.166
YK4	2.355	0.613	14.743	0.000	10.540	3.168	35.074
Positive margin	1.906	0.398	22.954	0.000	6.725	3.084	14.665

Cox Proportional Hazards Model for the risks of lymph node metastasis, primary site recurrence, distant metastasis, and OSCC death. ^1^4c or 4d of the histopathologic classification of oral squamous cell carcinoma according to Yamamoto et al., 1983 (10). ^2^Major width of the tumor was categorized into >30 mm and ≤30 mm. ^3^ T3 to T4 of tumor (T) category according to the International Union Against Cancer (UICC) TNM classification of malignant tumors of the lip and oral cavity. ^4^Histological tumor positive of the surgical margin. ^5^Death outcome from uncontrollable oral squamous cell carcinoma. Continuous variables of gene expression ratios are categorized according to the cut off points introduced by receiver operating characteristic curves. Median of ITGA3/CD9 and higher 30% group of ITGB4/JUP are used for categorization. B, regression coefficient; SE, standard error; OR, odds ratio; 95% CI, 95% confidence interval.

### Primary site recurrence

Regarding primary site recurrence (PSR), the Cox proportional hazards model found high-ITGA3/CD9, T3-4 (TNM category) and positive margin to be independently significant variables (Table 3B). Although the positive margin is a sequential clinical event following surgery, it was involved in this analysis because of its considerable influence on PSR. The rate of PSR was represented by the K-M curves (one minus cumulative survival) between groups of [high-ITGA3/CD9 and T3-4]-positive cases and the remaining (negative) cases according to marginal status strata (Figure 2b). Risk of PSR was clearly enhanced in the positive group, indicating that positive margin is a remarkable causal event of PSR especially in [high-ITGA3/CD9 and T3-4]-positive cases.

### Distant metastasis

In the Cox proportional hazards model on distant metastasis (DM), high-ITGB4/JUP and ITGA3/CD9 levels were reported as independently significant variables (Table 3C). In contrast to other clinical events, parameters related to tumor expansion, histopathological parameters, and LNM did not exhibit a significant influence in multivariate analysis, while high-ITGB4/JUP exhibited the strongest influence. The rate of DM was presented by the K-M curves (one minus cumulative survival) between the groups of [high-ITGA3/CD9 and high-ITGB4/JUP]-positive cases and remaining negative cases according to size category strata (Figure 2c). Among the 183 cases comprising “size ≤30 mm”, all of the nine cases that developed DM were extracted in 39 cases by a [high-ITGA3/CD9 and high-ITGB4/JUP]-double positive status. Among all 270 cases, 12 out of a total of 14 that developed DM were detected by a high-ITGA3/CD9 and high-ITGB4/JUP double-positive status. Both false positive cases had a tumor size of over 35 mm, suggesting a higher diagnostic reliability for [high-ITGA3/CD9 and high-ITGB4/JUP] status in early OSCC.

### Death outcome from uncontrollable OSCC

Regarding the OSCC death outcome (DO) Cox proportional hazards model, high-ITGA3/CD9, YK4, and positive margin were reported as independently significant variables (Table 3D). The cumulative survival was represented by the K-M curves between the groups of [high-ITGA3/CD9 and YK4]-positive cases and the remaining negative cases according to marginal status strata (Figure 2d). The risk of OSCC death was significantly higher in [high-ITGA3/CD9 and YK4]-positive cases, in which positive margin was a lethal treatment consequence in clinical outcome.

## Discussion

There are two types of OSCC metastatic trait. The first is simple lymph node metastasis (LNM) that can be locoregionally controlled, and the second is characterized by uncontrollable locoregional dissemination as well as distant metastasis (DM) through the blood circulation, leading to death. Distinguishing these two types of metastasis is difficult by current diagnostic procedures. In our previous study, we analyzed the ratio of expression of the 11 ITG family genes to that of the 14 functionally related genes; in total, 154 gene expression ratios for 66 tongue SCC cases [15]. We also investigated the potential of 45 tetraspanin family gene expression ratios that were calculated based on 6 tetraspanin family genes with housekeeping functionality or functionally related genes for 73 gingival SCC cases [16]. The results of these prior studies revealed two ITG gene expression ratios–those of ITGA3/CD9 and ITGB4/JUP–as candidate biomarkers for OSCC.

Gene expression analysis using the entire tumor tissue is expected to involve several biases depending on cell composition, due to choice of sampling site, and degradation of molecules. Biopsy samples inevitably contain cell populations comprising cancer cells, cancer stroma cells such as fibroblasts, and inflammatory cells, and in some cases normal epithelial cells. However, we did not want to limit our analysis to the cancer cell population because we believe that analysis of the whole biopsy sample is essential for collecting practical information on the overall aspects of cancer biology that may contribute to the clinical behavior of the disease. For these reasons, we have focused on devising diagnostic gene expression ratios that are not affected by the contamination of normal cells or by sampling biases. To address this issue of biases, we have adopted a functional referencing strategy that uses gene expression data obtained by calculating gene pairs with relevance to intercellular localization and/or molecular function. As the consequence, we have demonstrated the practical benefits of ITGA3/CD9 and ITGB4/JUP in this study.

ITGA3/CD9 levels represent biological traits associated with lymphatic dissemination and local invasiveness. K-M curves for LNM showed that a [high-ITGA3/CD9 and YK4] status can identify highly metastatic cancer capable of early lymph node invasion (Figure 2a). High-ITGA3/CD9 and T3-4 were also reported to be related to primary site recurrence (PSR) (Table 3B and Figure 2b), and positive margin is the most significant factor of PSR. However, only 42% of positive margin cases resulted in PSR, implying that biological traits are also critical in PSR. The α3β1 integrin complex is a major receptor for laminin 5 [17] and is involved in the maintenance of epithelial integrity, cell proliferation and motility, and survival of migrating keratinocytes through adhesion to extracellular matrix components [21-23]. CD9 is a tetraspanin family molecule, which forms tetraspanin webs (tetraspanin-enriched microdomains) by associating with various partner molecules such as integrins, growth factor receptors, and other tetraspanin molecules, to affect cell adhesion, signal transduction, proliferation, motility, differentiation, and cancer metastasis [16,24-28]. It has been reported that CD9 negatively influences cancer cell motility by regulating the re-organization of the actin cytoskeleton [29]. Collectively, it could be hypothesized that the ITGA3/CD9 gene expression ratio reflects the phase of cell motility and invasion in OSCC tissue.

In contrast to LNM and PSR, the ITGB4/JUP status exhibited a peculiar contribution for the prediction of distant metastasis (DM) (Table 3C), implying the involvement of a distinctive biological mechanism in DM. It is also characteristic of DM that no clinicopathological parameters were reported as contributing factors. These findings suggest the intervention of a critical step that depends on the function of the integrin β4 subunit in the process of DM. α6β4 integrin is a transmembrane component of hemidesmosomes and functions mainly as a receptor for laminin 5 [30]. Plakoglobin (*JUP*) is a component of the attachment plaque lining the cytoplasmic side of the desmosome to anchor intermediate filaments [31,32]. Since both the integrin β4 subunit and JUP colocalize around the cell membrane, and mediate functions through cell adhesion, their expression ratio may reflect the oncological phase of SCC cells.

DM is rather rare in cancer-bearing conditions, despite the continuous release of numerous cancer cells into the circulation. This may be because most circulating cancer cells die without proliferating even after being implanted into distant tissues. In normal conditions, epithelial cells detached from the matrix or those that are attached via the wrong molecules undergo anchorage-related apoptosis. Therefore, acquirement of the ability for anchorage-independent survival, migration, and growth is essential for isolated tumor cells to engage in the process of hematogenous metastasis. Expression of integrin β4 has been associated with tumor progression, aggressive behavior and poor prognosis in human malignant neoplasms [15,33-36]. It is reported that α6β4 integrin contributes to anchorage-independent growth through the ERK1/2 signaling pathway and to invasion through the combined activation of PI3K and Src [37]. Aberrant cytoplasmic localization of integrin β4 in highly invasive OSCC cells suggests acquired anchorage-independent growth and motility through impaired *ITGB4* expression [15].

## Conclusions

**Figure 3:**
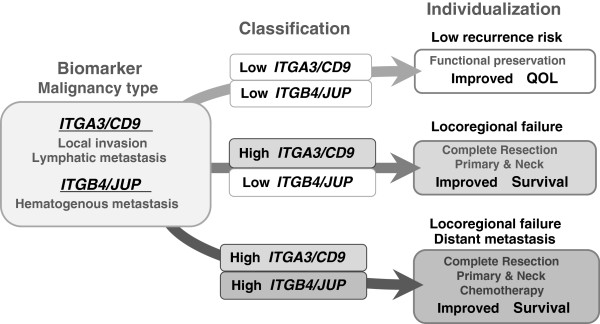
A hypothetical biomarker-oriented individualization of oral squamous cell carcinoma (OSCC) treatment based on the early diagnosis of OSCC malignancy type.

The biomarker system of the ITGA3/CD9 and ITGB4/JUP expression ratios may enable us precisely estimate the extent of local invasion and lymphatic metastasis, or hematogenous dissemination of OSCC. Information on ITGA3/CD9 level should enable surgeons to use an appropriate resection procedure to minimize the incidence of local recurrence as well as improve patient QOL by reducing oral dysfunction after treatment. The ITGB4/JUP level also provides information on the risk of distant metastasis, enabling effective pre- or postoperative adjuvant therapies to be given before metastatic lesions manifest (Figure 3). Recent genome-wide sequence studies have provided evidence that head and neck SCCs (HNSCCs), although morphologically similar, constitute distinct diseases at the molecular level. Since the major driver mutations accompanied by a large variety of genetic alternations are implicated in the carcinogenesis of SCC, it is thought to be impossible to assess malignancy type using only a few genetic markers. No genetic disruption in ITGA3, ITGB4, CD9, or JUP genes has been identified in reported HNSCC cases [38]. Therefore, changes in the ITGA3/CD9 and ITGB4/JUP levels as phenotypes due to a variety of mutations may serve as common indicators of biological malignancy of SCC. Further prospective clinical study will be indispensable for verifying the validity and clinical reliability of using gene expression ratios as a diagnostic means for distinguishing potential lymphatic and hematogenous disseminations. Likewise, biological involvement of the ITG molecules in locoregional invasion and hematogenous dissemination of OSCC remain to be determined.

## Abbreviations

OSCC: Oral squamous cell carcinoma; QOL: Quality of life; RT-QPCR: Reverse transcription-quantitative real time polymerase chain reaction; LNM: Lymph node metastasis; PSR: Primary site recurrence; DM: Distant metastasis; DO: Death outcome; K-M curve: Kaplan-Meier curve.

## Competing interests

There are no competing interests to declare.

## Authors’ contributions

MN, AAN, TKo, and KU carried out gene expression analysis and immunohistochemistry. MN, KU, TK, HH, KT, HK, NI, TO, and MO took charge of acquisition of the tumor sample and clinical data. MN and NK participated in the design of the study and performed statistical analysis. MN, KS, HK, and SS summarized and interpreted the data. MN wrote the paper; KS, RT, and TKa were involved in critically revising the manuscript for important intellectual content. All authors have read and approved the final manuscript.

## Pre-publication history

The pre-publication history for this paper can be accessed here:

http://www.biomedcentral.com/1471-2407/13/410/prepub
